# Characterisation of inorganic constitutions of condensate and solid residue generated from small-scale ex situ experiments in the context of underground coal gasification

**DOI:** 10.1007/s11356-021-15780-8

**Published:** 2021-08-07

**Authors:** Sivachidambaram Sadasivam, Renato Zagorščak, Hywel Rhys Thomas, Krzysztof Kapusta, Krzysztof Stańczyk

**Affiliations:** 1 Geoenvironmental Research Centre (GRC), School of Engineering, Cardiff University, The Queen’s Buildings, The Parade, Cardiff, CF24 3AA UK; 2 Główny Instytut Górnictwa (Central Mining Institute), Plac Gwarków 1, 40-166 Katowice, Poland

**Keywords:** Underground coal gasification, Condensate, Residue, Inorganic characterisation

## Abstract

This paper deals with the characterisation of inorganic constitutions generated at various operating conditions in the context of underground coal gasification (UCG). The ex situ small-scale experiments were conducted with coal specimens of different rank, from the South Wales Coalfield, Wales, UK, and Upper Silesian Coal Basin, Poland. The experiments were conducted at various gaseous oxidant ratios (water: oxygen = 1:1 and 2:1), pressures (20 bar and 36 bar) and temperatures (650°C, 750°C and 850°C). Increasing the amount of water in the oxidants proportionately decreased the cationic elements but increased the concentrations of anionic species. The temperature played minor impact, while the high-pressure experiments at temperature optimum to produce methane-rich syngas (750°C) showed significant reduction in cationic element generation. However, both coal specimens produced high amount of anionic species (F, Cl, SO_4_ and NO_3_). The “Hard” bituminous coal from Poland produced less gasification residues and condensates than the South Wales anthracitic coal due to its higher reactivity. The inorganic composition found in the solid residue was used in the theoretical calculation to predict the dissolved product concentrations when the solid residue interacts with deep coal seam water in the event of UCG cavity flooding. It was evident from the solubility products of the Cr, Ni and Zn that changes in the groundwater geochemistry occur; hence, their transportation in the subsurface must be studied further.

**Supplementary Information:**

The online version contains supplementary material available at 10.1007/s11356-021-15780-8.

## Introduction

Underground coal gasification (UCG) has been studied in situ to explore the feasibility of gasifying unmineable coal seams (Perkins 2018). The field trials conducted at shallow level with operating pressure higher than the hydrostatic pressure showed that high level of contamination escaped to the groundwater environment (Australian government’s environmental protection notice 2016; Gemmell 2016). The UCG operations at deeper coal seams appear to be viable due to the possibilities of increasing the operation pressure to get optimum production of syngas with high-value components and reduced threat of contamination transport. There are presumptions that operating deeper than the groundwater table and below the impermeable barriers would minimise the impact on groundwater. However, the UCG trial conducted at El Tremedal (550 m deep) showed water ingression to the cavity, and the produced water has been characterised as toxic (Sury et al. 2004). There is limited information on the characterisation of groundwater/produced water from the UCG conducted at deeper coal seams. To address the issue, contaminations quantified from the experiments conducted at laboratory scales can be used as a benchmark to understand the impact on the groundwater and the rock strata, which is the focus of present research.

Many studies addressed the contaminants generated from UCG, and the main interest was focused on organic contaminants such as phenol, benzene, toluene, ethylbenzene and xylene (Smoliński et al. 2012). Likewise, inorganic contaminants would have an impact on the groundwater and cap rock-water interaction. So, the present study focuses on the quantitative characterisation of the inorganic constitutions of condensate and gasification residues generated from small-scale laboratory experiments. The major inorganic constitutions found in and near UCG cavity are sodium (Na^+^), calcium (Ca^2+^), sulphate (SO_4_^2-^), bicarbonate (HCO_3_^-^), chlorine (Cl^-^), ammonia (NH^3+^), fluoride (F^-^) and bromide (Br^-^) (Humenick and Mattox 1978; Campbell et al. 1979; Liu et al. 2007; Kapusta and Stańczyk 2011; Kapusta et al. 2013). More volatile trace elements transported by gas phase emission during UCG could condensate in the cooler parts of the reactors, and less volatile trace elements such as Ni and Cr can be found in the solid ash (Liu et al. 2006a; Liu et al. 2006b). The emission of hazardous trace elements along with the gases would primarily affect the gas cleaning process and would have an impact on the components of the fuel cells if integrated with gasification (Yoshiie et al. 2011). The escaping contaminants from the UCG cavity would cause change in concentrations of major ions in the groundwater and influence the rock-water equilibrium. Consequently, the equilibrium shift might influence the rate of rock dissolution. Further, the ash left in the cavity contains high concentrations of inorganic elements (Sadasivam et.al., 2020a). The dissolution of inorganic constitutions from the ash left in the cavity would moderately affect the groundwater quality near the cavity. The gas cleaning process during the UCG also produces effluent with inorganic metals, nonmetals and metalloids. The quantity of contaminants generated was correlated with the operating conditions and coal ranks. The condensates generated at high-pressure operating conditions exhibited lower concentrations of inorganic species than the experiments conducted at atmospheric pressure (Sadasivam et al. 2020a). “Hard” coal specimens released significantly higher concentration levels of inorganic contaminants than the lignite coal (Kapusta and Stańczyk 2015).

The current study characterised the inorganic constitutions of the condensate and the solid residue generated from two coals of different rank gasified at various operating conditions in terms of pressure, temperature and oxidants ratios. Additionally, the inorganic species were studied theoretically for their solubility product species concentrations when the solid residue interacts with coal seam water to understand the impact of gasification residues on the groundwater and subsurface.

## Materials and methods

The coal specimens used in the study were procured from South Wales Coalfield, UK (referred as “Six Feet”) and Upper Silesian Coal Basin, Poland (referred as “Hard” coal). The properties of the two coal specimens are presented in Table 1.

**Table 1:** Proximate and ultimate characteristics of the coal specimens used for the gasification tests

**No.**	**Parameter**	**Coal sample**	
		**“Six Feet”**	**“Hard” coal**
**As received**			
1	Total moisture W_t_^r^, %	1.15 ± 0.40	3.60 ± 0.40
2	Ash A_t_^r^ , %	4.61 ± 0.30	8.74 ± 40
3	Volatiles V^r^, %	9.92 ± 0.12	27.67 ± 0.50
4	Total sulphur S_t_^r^, %	1.55 ± 0.04	0.31 ± 0.02
5	Calorific value Q_i_^r^, kJ/kg	33,416 ± 220	28,798 ± 200
**Analytical**			
6	Moisture W^a^, %	0.84 ± 0.30	2.18 ± 0.27
7	Ash A^a^, %	4.62 ± 0.30	8.87 ± 0.63
8	Volatiles V^a^, %	9.95 ± 0.13	28.08 ± 0.92
9	Heat of combustion Q_s_^a^, kJ/kg	34,414 ± 228	30,317 ± 161
10	Calorific value Q_i_^a^, kJ/kg	33,527 ± 221	29,258 ± 201
11	Total sulphur S^a^, %	1.55 ± 0.04	0.31 ± 0.08
12	Carbon C_t_^a^, %	87.31 ± 0.66	75.35 ± 1.13
13	Hydrogen H_t_^a^, %	3.97 ± 0.28	4.61 ± 0.40
14	Nitrogen N^a^, %	1.29 ± 0.12	1.20 ± 0.22
15	Oxygen O_d_^a^, %	0.50 ± 0.05	7.65 ± 0.1
16	Specific gravity, g/cm^3^	1.35 ± 0.028	1.40 ± 0.018

**Figure 1:**
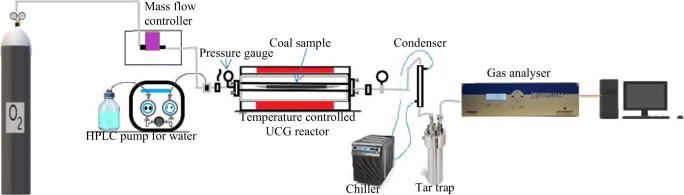
Schematic of bespoke small-scale ex situ UCG experimental simulation rig

A bespoke experimental rig was used to perform the gasification tests. The experimental rig (Figure 1) can operate at maximum of 50 bar pressure and 900°C temperature. Experiments were carried out at 36 bar and 20 bar pressure conditions and at 650 °C, 750 °C and 850 °C temperature settings for 90 min. Mixture of H_2_O and O_2_ was used as the oxidants. The flow rates of oxidants were adjusted to keep the molar ratio of the H_2_O and O_2_ at 1:1 (H_2_O:O_2_) and 2:1. The matrix of the operational procedure (pressure, temperature and oxidants flow rates) is explained in Table 2, and further details on the experimental setup and procedures have been described in Sadasivam et.al. (2020b).

**Table 2:** Summary of operating conditions and measurements of mass of the post-gasification solid residues and volume of the condensate obtained for the tests conducted on “Six Feet” coal (South Wales, UK) and “Hard” coal (Silesia, Poland)

**Test No.**	***T*** **(°C)**	***P*** **(bar)**	**H**_**2**_**O:O**_**2**_ **ratio**	**Mass of the gasification solid residue (g)**		**Volume of condensate (mL)**	
				**“Six Feet” coal**	**“Hard” coal**	**“Six Feet” coal**	**“Hard” coal**
**1**	650	20	1:1	12.51	9.02	10	12
**2**	650	20	2:1	11.13	9.33	72	30
**3**	850	20	1:1	8.18	4.29	8	28
**4**	850	20	2:1	7.92	5.35	20	32
**5**	650	36	1:1	16.52	2.90	52	10
**6**	650	36	2:1	8.16	16.78	108	50
**7**	850	36	1:1	13.21	14.28	11	10
**8**	850	36	2:1	13.2	11.27	70	128
**9**	750	20	2:1	11.51	6.64	78	40
**10**	750	36	2:1	18.18	14.06	130	58

Each experiment was carried out with 30 g of intact coal specimens. Deionised water was used in the experiments to estimate the inorganic ions generated in the UCG reaction. The condensates from the UCG experiments were collected in the tar trap (Figure 1). The volume of condensates and mass of the coal residue were measured after each experiment (Table 2). The collected condensates had been stored at 4°C until the chemical analysis. The pH and EC (electric conductivity) were measured using Mettler Toledo SevenMulti pH/EC meter, and the alkalinity was measured by titration method as described in APHA (2005).

The condensates were filtered to remove the tar and digested using con. HNO_3_ prior to analysing in a Perkin Elmer Optima 2100 inductively coupled plasma optical emission spectrometry (ICP-OES) for the inorganic cations. The major anions were measured using a Dionex ICS 2000 ion chromatography configured with hydroxyl column.

The gasification residue specimens were digested using an acid mixture of HF, HNO_3_, HCL and H_3_BO_3_ in an Anton Parr Multiwave 3000 microwave digestion system. The resulting solution was analysed for the concentrations of cationic elements using ICP-OES, and the results were calculated back to represent the concentration in the solid residue.

Computer-based geochemical application software Geochemist’s Work Bench (GWB) was used to calculate the solubility product of Fe, Al, Ni, Cr and Zn species when the residue interacts with groundwater from the South Wales Coalfield. The React programme of GWB was used to calculate the solubility product concentrations when 1 g of the residue (average values of the residue generated by “Six Feet” coal, Table 3) interacts with 1 kg of groundwater (Table 3). The pH value was slid from the natural pH (8.88) value of the groundwater to 2.5 and to 11.5.

**Table 3:** Concentrations and method used in the gasification residue-groundwater interaction to calculate concentrations of the solubility products

**Concentration of species in 1 kg of groundwater (measured from a coal seam water, South Wales Coalfield)**	**Amount of species in 1 g of gasification residue (average value of the elements in the gasification residue generated from “Six Feet” coal from South Wales Coalfield, converted to oxide equivalents)**
O_2(aq)_ = 2.5 mg/kg	16.37 mg of Na_2_O
pH = 8.88	24.7 mg of CaO
HCO_3_^-^ = 656.6 mg/kg	5.02 mg of MgO
NO_3_^-^ =.02 mg/kg	1.5 mg of K_2_O
F- = 2.44 mg/kg	248.3 mg of Al_2_O_3_
Cl- = 79.8 mg/kg	0.35 mg of MnO
SO_4_^--^ = 24.33 mg/kg	14.21 mg of BaO
Fe^++^ = .09 mg/kg	1.98 mg of Cr_2_O_3_
Na^+^ =540 mg/kg	7.21 mg of NiO
Ca^++^ = 2.87 mg/kg	2.75 mg of SrO
Mg^++^ =1.29 mg/kg	3.32 mg of TiO_2_
K^+^ = 10.03 mg/kg	10.37 mg of ZnO
Mn^++^ = .07 mg/kg	21.65 mg of Fe_2_O_3_
H_2_AsO_4_^-^ = .18 mg/kg	Method: the React programme in the GWB and the thermodynamic data base supplied along with GWB was used in the calculations. The groundwater concentrations were used to set the input constrains. The average value of elements was converted into oxides and input as simple oxide reactants in React programme. The pH was slid from 8.88.
B(OH)_3(aq)_ = 0.86 mg/kg	
Cd^++^ = .001 mg/kg	
CrO_4_^--^ = .022 mg/kg	
Cu^++^ = 0.003 mg/kg	
MoO_4_^--^ = 0.017 mg/kg	
Ni^++^ = 0.01 mg/kg	
Pb^++^ = .02 mg/kg	
Sb(OH)_3(aq)_ = 0.014 mg/kg	
SeO_3_^--^ = 0.11 mg/kg	
Ti(OH)_4(aq)_ = 0.005 mg/kg	
Zn^++^ =0.01 mg/kg	
Sr^++^ = 0.1 mg/kg	
Al^+++^ = 0.001 mg/kg	
Ba^++^ = 0.001 mg/l	

## Results and discussions

### Mass of the UCG solid residues and volume of the condensate

**Figure 2:**
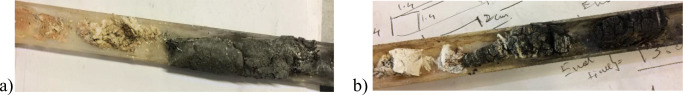
Gasification residues: (**a**) “Six Feet” coal and (**b**) “Hard” coal

Table 2 presents the summary of mass of the post-gasification residues and volume of the condensates obtained after the gasification of “Six Feet” coal (South Wales, UK) and “Hard” coal (Silesia, Poland). The amount of solid gasification residue and volume of condensate produced by “Hard” coal was lower than the “Six Feet” coal. The pore structure difference between coals of different rank has an impact on the gasification and eventually the amount of ash produced (Kim et al. 2011; Mishra et al. 2018). Due to heterogenous structure of lower-rank coals, containing macropores, mesopores and micropores, more water can penetrate into the coal structure to participate in reactions. So, the solid residues generated with “Hard” coal were lower than the “Six Feet” coal which implies that more coal was consumed during the gasification of Silesian “Hard” coal due to its higher reactivity (Figure 2). The “Six Feet” anthracitic coal from South Wales Coalfield produced more methane, hydrogen and carbon monoxide than the “Hard” bituminous coal, owing to its higher effective carbon content during the stable gas production phase (Sadasivam et al. 2020b). The volume of water condensate reflects this, as the methane-generating reactions (CO+3H_2_→CH_4_+H_2_O, CO_2_+4H_2_→CH_4_+2H_2_O) indicate more water condensate generation with high-rank coals, and the unreacted water (due to the lower reactivity of the high-rank coal) also adds up to the excess water generated during the gasification of “Six Feet” coal. This demonstrates that the difference in the coal rank plays a major role in the amount of coal involved in the reactions generating high-value syngas components.

### UCG post-processing condensed matter characterisation

**Figure 3:**
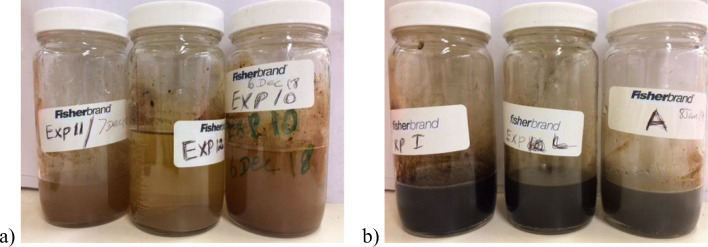
Condensed matter: (**a**) “Six Feet” coal and (**b**) “Hard” coal.

From the post-gasification condensed matter collected from the tar trap, as shown in Figure 3, it is visibly apparent that the “Hard” coal produced more tar content than the “Six Feet” coal specimen. It can be explained by the higher content of volatiles of the “Hard” coal sample, i.e. 27.67% compared to 9.92% for the “Six Feet” coal. The results of pH, electric conductivity (EC) and alkalinity characterisation analysis obtained on the post-gasification condensed matter of “Six Feet” coal (South Wales, UK) and “Hard” coal (Silesia, Poland) are given in Tables 4 and 5, respectively. The complete results of the analysis are given in tables in the supplementary data. The pH of the condensates generated at lower temperature experiments (test 1, test 2, test 5 and test 6, at 650°C) were in acidic condition and independent of the pressure. Comparing the pH values with the contents of anions presented in Figure 5, correlation between acidic anion SO_4_^2-^ and pH value (negative correlation) is apparent. The data show that more SO_4_^2-^ ions were observed at the lower gasification temperature. Possibly, sulphur may have evaporated at higher temperatures and formed gaseous compounds that were transported along with UCG gas. For example, test numbers 5 and 6 that were conducted lower temperature showed higher SO_4_ ion concentration, relatively low bicarbonate ion concentration and acidic pH. The trend is reversed at higher temperature and is reflected by the pH value. Comparing both coals, “Hard” coal produced higher dissolved ion concentration which led to its higher EC value.

**Table 4:** Parameters determined in the condensed matter obtained from tests conducted on “Six Feet” coal (South Wales, UK)

**Parameter**	**Test 1**	**Test 2**	**Test 3**	**Test 4**	**Test 5**	**Test 6**	**Test 7**	**Test 8**	**Test 9**	**Test 10**
**pH**	2.43	2.58	8.60	Nm	5.22	5.56	8.30	7.80	6.79	6.78
**EC (mS/cm)**	8.99	9.11	9.00	Nm	8.51	5.05	15.00	13.86	8.45	6.54
**Total alkalinity as CaCO**_**3**_ **(mg/L)**	Nm	Nm	18,100	Nm	210	230	20,050	6670	2030	2620

**Table 5:** Parameters determined in the condensed matter obtained from tests conducted on “Hard” coal (Silesia, Poland)

**Parameter**	**Test 1**	**Test 2**	**Test 3**	**Test 4**	**Test 5**	**Test 6**	**Test 7**	**Test 8**	**Test 9**	**Test 10**
**pH**	7.39	6.60	8.09	7.79	Nm	5.84	7.93	6.89	5.99	6.92
**EC (mS/cm)**	13.08	8.70	19.10	23.05	Nm	7.66	19.50	9.40	11.10	11.40
**Total alkalinity as CaCO**_**3**_ **(mg/L)**	2220	1460	7320	8980	Nm	720	8380	3160	660	3600

**Figure 4:**
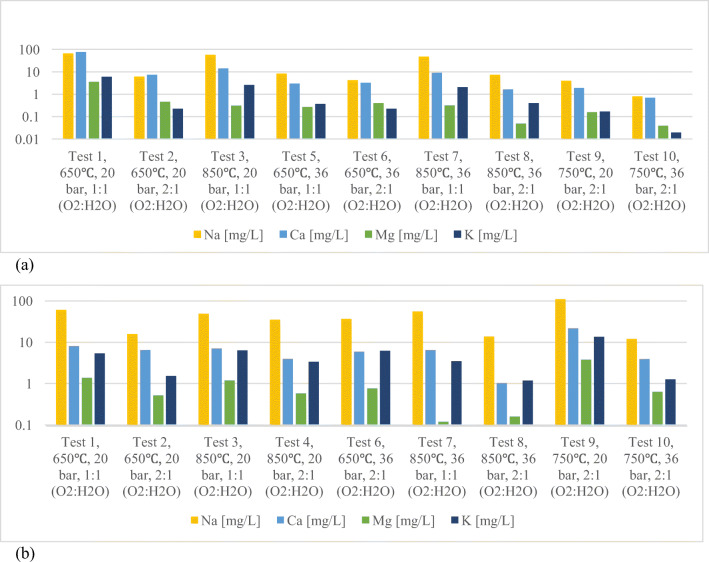
Variation of the major cations’ concentrations upon experimental conditions for (**a**) “Six Feet” and (**b**) “Hard” coal

**Figure 5:**
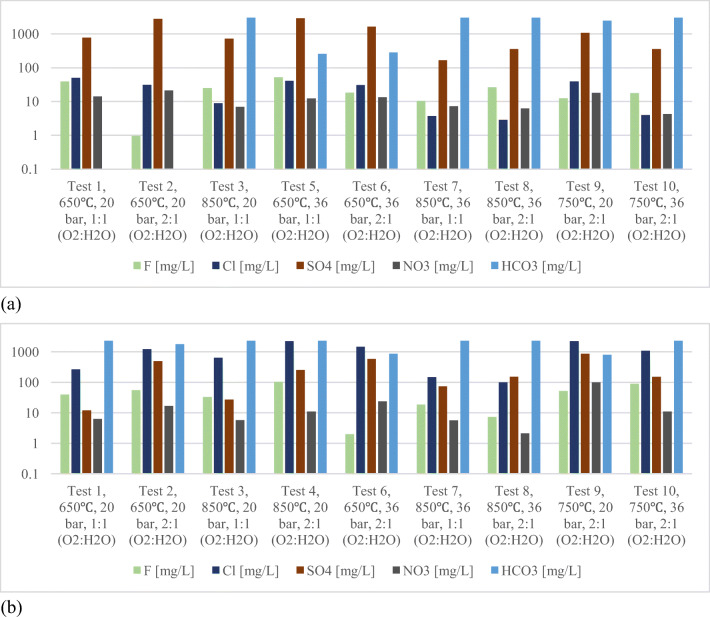
Variation of the major anions’ concentrations upon experimental conditions for (**a**) “Six Feet” and (**b**) “Hard” coal

**Figure 6:**
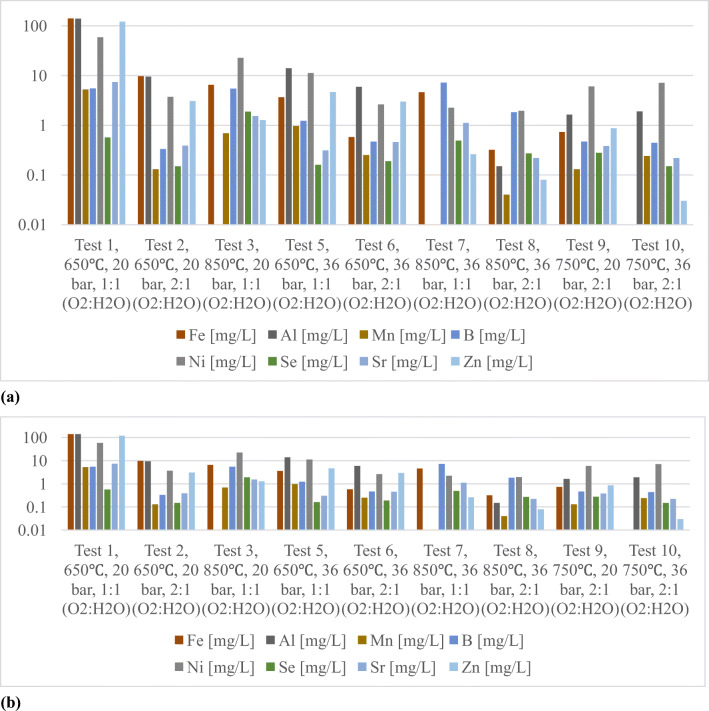
Concentrations of minor ions upon varying experimental conditions for (**a**) “Six Feet” and (**b**) “Hard” coal condensates

Figures 4 and 5 show the concentrations of major cations (Na, K, Ca and Mg) and anions (F, Cl, SO_4_ and NO_3_) in the condensate generated from both coals, respectively. In terms of cations, comparison of the results from tests 1, 3, 5 and 7 with 2, 4, 6 and 8 shows that the concentration levels were higher in experiments with 1:1 (H_2_O:O_2_) oxidant ratio than 2:1. In terms of temperature, tests 6 and 8 show that the temperature increase had minor impact and the oxidant ratio played a major role in contaminant generation. Experiments 9 and 10 with both coals clearly show the reduction in concentrations at 36 bar pressure compared to 20 bar pressure. This pattern has been reflected in other minor and trace elements as well (Figure 6). In terms of anions, the tests with 2:1 oxidant ratio generated more anions and the experiments with higher pressure and temperature marginally reduced the concentration levels of the major anions (Figure 5). The “Hard” coal released more Cl^-^ ions than the “Six Feet,” whereas for SO_4_^-^ ions, it is vice versa. The interpretation must not be confused with anion/cation balances as large number of organic complexes are present in the condensates.

Both coal specimens produced noticeable concentration levels (ppm levels) of Mn, Cu, B, Cr, Ni, Sr and Zn. The acidic nature of the condensates reflects on the concentrations of pH dependant solubility of few cations (example Zn) which are moderately higher than the condensates from higher temperature experiments. The Fe concentrations of “Hard” coal were much higher than the “Six Feet” coal. Considerable amount of Ni and Cr was found in condensate produced by both coal specimens. Higher concentrations of sulphate measured in the “Six Feet” coal condensates were the indications of hydrogeology of South Wales Coalfield (Farr et al. 2016). The pH and unsaturated nature of the elements in the condensate would affect the cap rock dissolution into the groundwater.

*Nm* not measured

*Nm* not measured

### Characteristics of UCG solid residues

As a certain amount of solid residue was required for chemical analysis, only several experiments produced the amount required to be considered for the analysis. In particular, post-gasification residues obtained from test 1, test 2 and test 3, test 4 and test 9 on “Six Feet” coal and post-gasification residues obtained from test 1, test 3, test 4 and test 8 on “Hard” coal were analysed for concentrations of particular elements. The results of the analysis are presented in Table 6.

**Table 6:** Parameters determined in post-gasification solid residues obtained from tests conducted on “Six Feet” coal (South Wales, UK) and “Hard” coal (Silesia, Poland)

**Parameter**	**“Six Feet” gasification residue (mg/g)**					**“Hard” gasification residue (mg/g)**			
	**Test 1**	**Test 2**	**Test 3**	**Test 4**	**Test 9**	**Test 1**	**Test 3**	**Test 4**	**Test 8**
**Na**	3.85	4.23	20.89	15.89	16.25	20.17	34.01	7.95	20.69
**Ca**	18.23	18.41	13.31	18.21	20.70	173.99	50.42	198.76	60.74
**Mg**	1.43	0.69	3.78	4.75	4.56	71.60	22.15	87.80	21.88
**K**	1.49	0.78	1.68	1.19	1.01	3.20	4.17	0.89	4.33
**Fe**	9.53	4.54	10.90	21.74	28.99	89.29	34.49	128.65	31.81
**Al**	137.69	123.99	124.39	147.33	156.40	30.33	129.24	19.02	124.04
**Mn**	0.10	0.06	0.37	0.48	0.34	2.57	0.66	3.45	1.18
**Ba**	6.44	41.30	4.44	5.42	5.86	1.41	2.77	1.05	2.19
**Be**	0.02	0.02	0.02	0.01	0.01	Nd	0.02	Nd	0.01
**Cd**	0.02	0.02	Nd	Nd	0.00	Nd	Nd	Nd	Nd
**Co**	0.34	0.12	0.09	0.11	0.11	Nd	0.13	Nd	0.09
**Cr**	0.83	0.67	2.33	1.24	1.72	0.97	0.53	0.48	0.37
**Cu**	0.56	0.38	0.45	0.45	0.68	0.17	0.24	0.16	0.24
**Li**	0.35	0.39	0.11	0.16	0.21	0.06	0.07	0.09	0.26
**Mo**	0.07	0.05	0.13	0.10	0.16	0.05	0.08	0.09	0.07
**Ni**	2.28	1.99	12.27	3.70	8.16	0.91	1.99	1.46	1.53
**Pb**	0.38	0.16	0.79	0.21	0.75	0.10	0.20	0.03	0.16
**Sb**	0.02	0.02	0.04	0.02	0.04	Nd	Nd	Nd	0.01
**Se**	0.01	0.02	0.05	0.03	0.04	0.01	0.01	Nd	0.01
**Sr**	4.09	3.97	0.72	0.90	1.17	0.61	0.32	0.53	0.25
**Ti**	1.23	1.15	2.62	1.97	3.04	0.48	1.35	0.34	1.42
**Tl**	0.02	Nd	0.03	0.02	0.01	0.02	0.01	0.02	0.01
**V**	1.04	0.33	0.19	0.24	0.38	Nd	0.29	Nd	0.23
**Zn**	5.93	7.91	18.82	4.71	4.46	2.27	3.24	1.46	1.75
**Mass % in ash**	19.60	21.12	21.84	22.89	25.51	39.82	28.64	45.22	27.33

*Nd* not detected

The mass percentage of elements in the residue shows that the “Hard” coal residue contains higher amount of inorganic constituents than the residue from “Six Feet” coal. Both coal specimens produced considerable amount of iron in the residue. The iron content in the residue can act as a sorbent for other species in the event of cavity flooding (Sadasivam et al. 2020a). On the other hand, the dissolution of elements in the residue would impact the surrounding groundwater quality. Considering the temperature range of the UCG operations, most of the elements in the residue would be in oxide forms. The solubility of most of the oxide phases depends on the pH.

**Figure 7:**
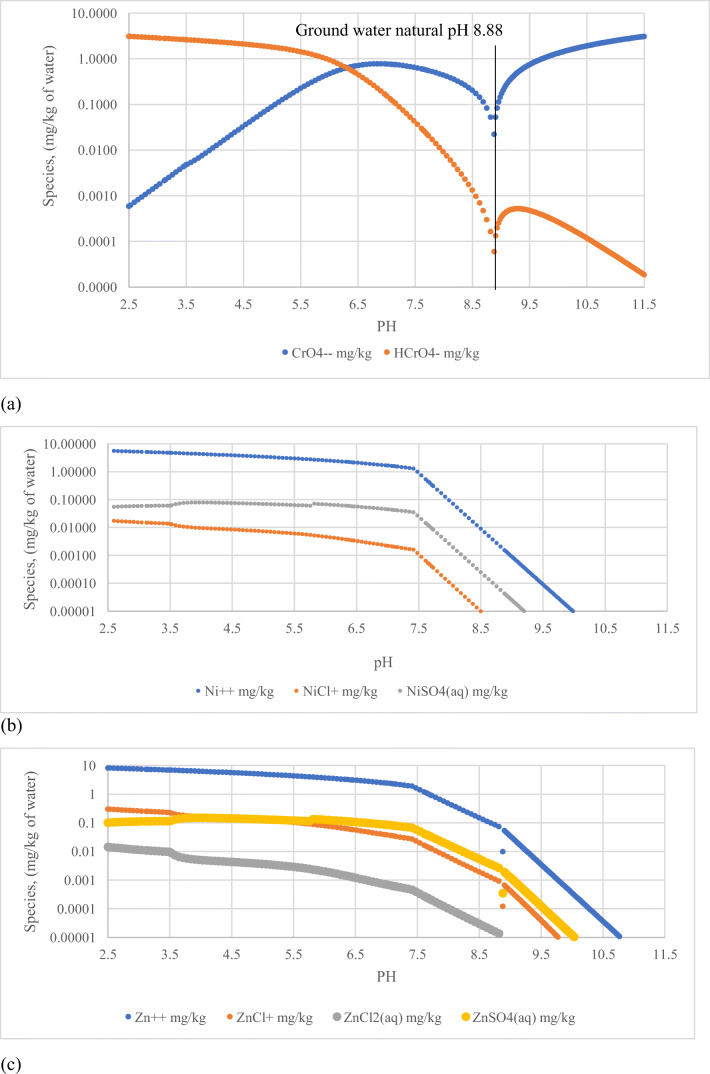
Concentrations of different species in 1 kg of groundwater after interacting with 1 g of “Six Feet” coal gasification residue at varying pH: (**a**) Cr, (**b**) Ni and (**c**) Zn

**Figure 8:**
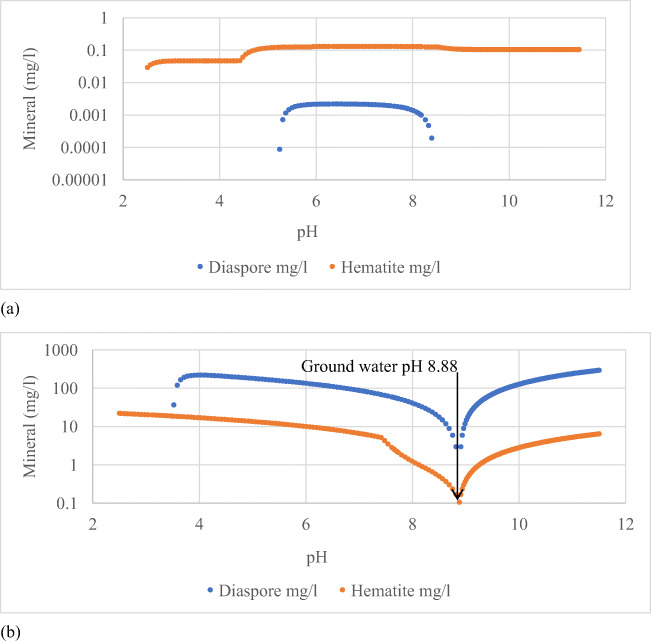
Concentrations of aluminium and hematite minerals in groundwater (**a**) before interacting with “Six Feet” residue and (**b**) after interacting with “Six Feet” residue upon varying pH from 8.88

Figure 7 shows the solubility product species of a few selected elements from Table 6. The average elemental composition of residue from the “Six Feet” coal in Table 6 was used in the determination of the theoretical mineral dissolution. The calculations were made based on the solubility of simple oxide form of the elements reacting with groundwater at 20°C. The inorganic chemical species present in the groundwater are shown in Table 3. The natural pH of the groundwater used in the calculations was 8.88. Figure 7 shows the concentrations of Cr, Ni and Zn species at varying pH in the groundwater, when 1 g of gasification residue interacts with 1 kg of groundwater. The scenario explains, for example, that the deep coal seam water with 0.01 mg/l Ni^++^ ion concentration would be increased to 1.3 mg/L by varying the pH from 8.88 to 7.4 because of the residue dissolution (Figure 7b). Cr dissolution from the residues produced tetrahedral chromate ions (CrO42-) in water. Acidification of aqueous chromate solution breaks down the equilibria (Butterworth-Heinemann 1997); HCrO_4_^-^ is formed as an intermediate compound at lower pH that dissociates at higher pH (Figure 7a). The Zn^+^ ion species dominates the Zn species in aqueous solutions with moderate chloride ion activity at varying pH conditions (McMahon et al. 2019; Ruaya and Seward 1986). The stability of zinc complexation with chloride is related to the chloride ion activity and thermodynamic stability of the complexation. The condition provided in the hypothetical dissolution of the gasification residue favours the Zn^+^ dominance in the groundwater than the uncharged ZnCl_2_ which could be dominant in saline system. The aluminium and iron were the predominant minerals in the residue. Figure 8 shows the diaspore (aluminium mineral) and hematite (iron mineral) minerals’ concentration changes in groundwater before and after interacting with the residue at various pH. This indicates that possible changes would occur in the groundwater geochemistry because of the residue dissolution when the UCG cavity floods.

## Conclusions

Observations made in the present study add to the current understanding of the inorganic contaminants generated from different types of coal and their environmental implications. The bituminous coal from Silesian Coal Basin, Poland, produced lower amount of gasification residue and lower volume of condensate than the anthracitic coal from South Wales Coalfield, which was the result of different reactivities of the coal specimens. Both coals produced condensates with acidic pH range at lower temperature (650°C). The oxidant ratio where the amount of water is more produced lower level of concentrations in the cationic species but high concentration of anionic species. Among the trace elements, noticeable concentrations of Mn, Cu, B, Cr, Ni, Sr and Zn were found, and “Hard” coal produced high amount of Fe in the condensates. While the temperature change had a minor impact on the contaminant generation, the tests with high pressure (36 bar) at temperatures (750°C and 850°C) showed considerably lower concentrations of ions in the condensates. Apart from the high amount of Al and Fe in the gasification residue, Cr, Ni and Zi were present in considerable amounts. The solubility product in the gasification residue water system showed the presence of active species of the above said elements at varying pH. Special attention needs to be focused on the activity of the species of the elements present in the gasification residue that would have an impact on the groundwater in the event of cavity flooding.

## Supplementary Information

ESM 1 (DOCX 50 kb)

## Data Availability

Not applicable.
