# Supplementary material for: Characterisation of inorganic constitutions of condensate and solid residue generated from small-scale ex situ experiments in the context of underground coal gasification
Source: Environ Sci Pollut Res Int. 2021 Aug 7;29(2):2203–13. doi: 10.1007/s11356-021-15780-8 (PMC8732842; doi:10.1007/s11356-021-15780-8)
Supplement: Supplementary file 1 — (DOCX 50 kb) [file 11356_2021_15780_MOESM1_ESM.docx]

**Supplementary data:**

The results of the inorganic chemical analysis of the condensate produced in the study are presented below.

**Table 1.** Parameters determined in the condensed matter obtained from particular tests conducted on “Six Feet” coal (South Wales, UK).

| **Parameter** | **Test 1** | **Test 2** | **Test 3** | **Test 4** | **Test 5** | **Test 6** | **Test 7** | **Test 8** | **Test 9** | **Test 10** |
| --- | --- | --- | --- | --- | --- | --- | --- | --- | --- | --- |
| **pH** | 2.43 | 2.58 | 8.60 | Nm | 5.22 | 5.56 | 8.30 | 7.80 | 6.79 | 6.78 |
| **EC [mS/cm]** | 8.99 | 9.11 | 9.00 | Nm | 8.51 | 5.05 | 15.00 | 13.86 | 8.45 | 6.54 |
| **Total alkalinity as CaCO_3_ [mg/L]** | Nd | Nd | 18100.00 | Nm | 210.00 | 230.00 | 20050.00 | 6670.00 | 2030.00 | 2620.00 |
| **Na [mg/L]** | 66.14 | 6.12 | 57.98 | Nm | 8.43 | 4.25 | 47.13 | 7.35 | 3.98 | 0.81 |
| **Ca [mg/L]** | 77.94 | 7.32 | 14.21 | Nm | 3.02 | 3.22 | 9.11 | 1.68 | 1.92 | 0.70 |
| **Mg [mg/L]** | 3.57 | 0.47 | 0.31 | Nm | 0.27 | 0.40 | 0.32 | 0.05 | 0.16 | 0.04 |
| **K [mg/L]** | 6.04 | 0.23 | 2.64 | Nm | 0.37 | 0.23 | 2.09 | 0.40 | 0.17 | 0.02 |
| **Fe [mg/L]** | 140.94 | 9.73 | 6.49 | Nm | 3.64 | 0.58 | 4.60 | 0.32 | 0.73 | 0.01 |
| **Al [mg/L]** | 140.03 | 9.46 | 0.00 | Nm | 13.92 | 5.95 | 0.00 | 0.15 | 1.62 | 1.90 |
| **Mn [mg/L]** | 5.26 | 0.13 | 0.69 | Nm | 0.97 | 0.25 | 0.00 | 0.04 | 0.13 | 0.24 |
| **Ag [mg/L]** | Nd | Nd | Nd | Nm | Nd | Nd | Nd | Nd | Nd | Nd |
| **As [mg/L]** | 0.32 | 0.10 | Nd | Nm | 0.14 | 0.08 | Nd | Nd | 0.06 | 0.05 |
| **B [mg/L]** | 5.50 | 0.33 | 5.47 | Nm | 1.23 | 0.47 | 7.14 | 1.82 | 0.47 | 0.44 |
| **Ba [mg/L]** | Nd | Nd | Nd | Nm | Nd | Nd | 0.11 | 0.13 | 0.11 | 0.04 |
| **Cd [mg/L]** | 0.25 | Nd | Nd | Nm | Nd | Nd | Nd | Nd | Nd | Nd |
| **Co [mg/L]** | 0.35 | Nd | Nd | Nm | Nd | Nd | Nd | Nd | Nd | Nd |
| **Cr [mg/L]** | 20.00 | 0.76 | 0.27 | Nm | 1.22 | 0.17 | Nd | Nd | 0.17 | 0.13 |
| **Cu [mg/L]** | 2.77 | 0.64 | 8.24 | Nm | 0.08 | 0.41 | 1.37 | 0.05 | 0.27 | Nd |
| **Li [mg/L]** | 0.69 | Nd | Nd | Nm | Nd | Nd | Nd | Nd | Nd | Nd |
| **Mo [mg/L]** | Nd | Nd | 2.40 | Nm | Nd | 0.23 | 1.36 | 0.33 | 0.17 | 0.13 |
| **Ni [mg/L]** | 58.91 | 3.72 | 22.65 | Nm | 11.23 | 2.63 | 2.25 | 1.95 | 6.02 | 7.13 |
| **Pb [mg/L]** | Nd | Nd | Nd | Nm | Nd | Nd | Nd | Nd | Nd | Nd |
| **Sb [mg/L]** | Nd | Nd | Nd | Nm | Nd | Nd | Nd | Nd | Nd | Nd |
| **Se [mg/L]** | 0.57 | 0.15 | 1.88 | Nm | 0.16 | 0.19 | 0.49 | 0.27 | 0.28 | 0.15 |
| **Sr [mg/L]** | 7.40 | 0.39 | 1.53 | Nm | 0.31 | 0.46 | 1.12 | 0.22 | 0.38 | 0.22 |
| **Ti [mg/L]** | Nd | 0.04 | Nd | Nm | Nd | Nd | Nd | Nd | Nd | Nd |
| **Tl [mg/L]** | 0.41 | 0.05 | Nd | Nm | Nd | Nd | Nd | Nd | Nd | Nd |
| **V [mg/L]** | 1.45 | 0.06 | 0.16 | Nm | Nd | Nd | Nd | Nd | Nd | 0.01 |
| **Zn [mg/L]** | 120.47 | 3.08 | 1.27 | Nm | 4.67 | 2.98 | 0.26 | 0.08 | 0.87 | 0.03 |
| **F [mg/L]** | 39.49 | 0.96 | 24.90 | Nm | 52.06 | 18.31 | 10.50 | 26.50 | 12.50 | 17.70 |
| **Cl [mg/L]** | 50.38 | 30.88 | 8.88 | Nm | 41.17 | 30.71 | 3.73 | 2.89 | 38.74 | 3.94 |
| **SO_4_ [mg/L]** | 771.00 | 2816.00 | 729.00 | Nm | 2898.00 | 1630.00 | 167.00 | 353.00 | 1066.00 | 353.00 |
| **NO_3_ [mg/L]** | 14.07 | 21.27 | 7.01 | Nm | 12.19 | 13.34 | 7.21 | 6.20 | 18.06 | 4.22 |
| *Nd – not detected*  *Nm – not measured* | | | | | | | | | |  |

**Table 2.** Parameters determined in the condensed matter obtained from particular tests conducted on “Hard” coal (Silesia, Poland).

| **Parameter** | **Test 1** | **Test 2** | **Test 3** | **Test 4** | **Test 5** | **Test 6** | **Test 7** | **Test 8** | **Test 9** | **Test 10** |
| --- | --- | --- | --- | --- | --- | --- | --- | --- | --- | --- |
| **pH** | 7.39 | 6.60 | 8.09 | 7.79 | Nm | 5.84 | 7.93 | 6.89 | 5.99 | 6.92 |
| **EC [mS/cm]** | 13.08 | 8.70 | 19.10 | 23.05 | Nm | 7.66 | 19.50 | 9.40 | 11.10 | 11.40 |
| **Total alkalinity as CaCO_3_ [mg/L]** | 2220.00 | 1460.00 | 7320.00 | 8980.00 | Nm | 720.00 | 8380.00 | 3160.00 | 660.00 | 3600.00 |
| **Na [mg/L]** | 60.86 | 15.70 | 48.99 | 35.29 | Nm | 37.06 | 56.11 | 13.83 | 118.58 | 12.14 |
| **Ca [mg/L]** | 8.13 | 6.46 | 7.14 | 4.00 | Nm | 5.93 | 6.46 | 1.03 | 21.87 | 3.94 |
| **Mg [mg/L]** | 1.39 | 0.52 | 1.20 | 0.58 | Nm | 0.77 | 0.12 | 0.16 | 3.80 | 0.64 |
| **K [mg/L]** | 5.34 | 1.53 | 6.36 | 3.36 | Nm | 6.23 | 3.51 | 1.19 | 13.53 | 1.27 |
| **Fe [mg/L]** | 52.75 | 30.02 | 13.49 | 20.04 | Nm | 59.14 | 3.41 | 0.02 | 121.10 | 2.43 |
| **Al [mg/L]** | 8.00 | 5.23 | 2.12 | 3.12 | Nm | 3.98 | 2.21 | 1.27 | 4.57 | 0.95 |
| **Mn [mg/L]** | 2.66 | 2.34 | 1.65 | 2.15 | Nm | 2.63 | 0.94 | 0.22 | 7.56 | 0.64 |
| **Ag [mg/L]** | Nd | Nd | Nd | Nd | Nm | Nd | Nd | Nd | Nd | Nd |
| **As [mg/L]** | 0.11 | 0.14 | Nd | 0.18 | Nm | 0.05 | Nd | 0.03 | 0.13 | 0.03 |
| **B [mg/L]** | 7.33 | 5.65 | 4.90 | 6.48 | Nm | 4.38 | 10.29 | 2.76 | 6.89 | 4.25 |
| **Ba [mg/L]** | Nd | 0.17 | Nd | 0.29 | Nm | 0.24 | Nd | 0.18 | 0.13 | 0.12 |
| **Cd [mg/L]** | Nd | Nd | Nd | Nd | Nm | 0.06 | Nd | Nd | 0.03 | Nd |
| **Co [mg/L]** | Nd | 0.07 | Nd | 0.04 | Nm | 0.17 | Nd | Nd | 0.49 | Nd |
| **Cr [mg/L]** | 8.45 | 2.75 | 2.83 | 2.75 | Nm | 5.60 | 0.34 | 0.16 | 14.50 | 0.61 |
| **Cu [mg/L]** | Nd | 0.37 | 0.10 | 0.07 | Nm | 0.37 | Nd | 0.02 | 0.31 | Nd |
| **Li [mg/L]** | Nd | Nd | Nd | Nd | Nm | 0.05 | Nd | Nd | 0.31 | Nd |
| **Mo [mg/L]** | Nd | 0.13 | 0.10 | 0.15 | Nm | 0.07 | Nd | 0.13 | 0.22 | 0.15 |
| **Ni [mg/L]** | 25.90 | 16.11 | 15.26 | 12.15 | Nm | 16.95 | 4.57 | 2.31 | 100.60 | 14.43 |
| **Pb [mg/L]** | Nd | 0.17 | Nd | 0.03 | Nm | 1.03 | Nd | Nd | 1.74 | 0.03 |
| **Sb [mg/L]** | Nd | Nd | Nd | Nd | Nm | Nd | Nd | Nd | 0.17 | Nd |
| **Se [mg/L]** | Nd | 0.38 | 0.55 | 0.51 | Nm | 0.23 | Nd | 0.24 | 0.38 | 0.35 |
| **Sr [mg/L]** | 0.26 | 0.18 | 0.12 | 0.17 | Nm | 0.13 | 0.25 | 0.11 | 0.34 | 0.12 |
| **Ti [mg/L]** | Nd | Nd | Nd | Nd | Nm | Nd | Nd | Nd | Nd | Nd |
| **Tl [mg/L]** | Nd | Nd | Nd | Nd | Nm | Nd | Nd | Nd | Nd | Nd |
| **V [mg/L]** | Nd | Nd | Nd | Nd | Nm | Nd | Nd | Nd | Nd | Nd |
| **Zn [mg/L]** | 2.51 | 3.72 | 0.13 | 0.99 | Nm | 16.08 | 0.63 | 0.01 | 39.53 | 0.59 |
| **F [mg/L]** | 40.00 | 55.00 | 33.00 | 104.00 | Nm | 2.00 | 18.63 | 7.29 | 52.00 | 89.00 |
| **Cl [mg/L]** | 269.00 | 1231.00 | 639.00 | 2220.00 | Nm | 1473.00 | 148.00 | 99.70 | 2244.00 | 1101.00 |
| **SO_4_ [mg/L]** | 12.00 | 491.00 | 27.00 | 257.00 | Nm | 588.00 | 74.00 | 152.00 | 878.00 | 151.00 |
| **NO_3_ [mg/L]** | 6.36 | 16.90 | 5.70 | 11.00 | Nm | 23.66 | 5.62 | 2.11 | 100.00 | 11.00 |
| *Nd – not detected*  *Nm – not measured* | | | | | | | | | |  |
